# Modified transforaminal epidural steroid injection combined with pulsed radiofrequency: an effective treatment measure for lumbar radiculopathy

**DOI:** 10.3389/fsurg.2025.1566661

**Published:** 2025-04-25

**Authors:** Qi Yan, Xianggu Zhong, Jiarong Li, Leyu Zhao, Junjie Niu, Dawei Song, Jinning Wang, Yun Teng, Tianyi Wu, Xiao Sun, Rui Chen, Shuangfei Wang, Jun Zou

**Affiliations:** ^1^ Department of Orthopedics Surgery, The First Affiliated Hospital of Soochow University, Suzhou, China; ^2^ Department of Orthopedics Surgery, Wuzhong People’s Hospital, Suzhou, Jiangsu, China; ^3^ Department of Orthopedics Surgery, Chenghang Hospital, Zhangjiagang, Jiangsu, China

**Keywords:** transforaminal epidural steroid injection (TFESI), pulsed radiofrequency (PRF), radicular pain, lumbar radiculopathy, lumbar disc herniation (LDH)

## Abstract

**Background:**

Lumbar disc herniation (LDH) is a prevalent condition encountered in the clinical diagnosis and management of spinal surgery. Certain people may experience excruciating radicular pain in the lower extremities. If these symptoms are not promptly alleviated, they may progressively deteriorate, ultimately resulting in radiating pain in the lower extremities, advancing neurological impairments, and potential trouble in standing, significantly impairing the patient's quality of life. Consequently, clinicians require an expedited and efficacious approach to address radicular discomfort resulting from lumbar radiculopathy and promptly reinstate the patient's normal functionality.

**Objectives:**

This study seeks to assess the effectiveness of a modified transforaminal epidural steroid injection (TFESI) in conjunction with pulsed radiofrequency (PRF) for treating lumbar radiculopathy using a retrospective analysis.

**Methods:**

Our study examined patients with unilateral lower limb radicular pain persisting for three months or more due to LDH, in whom conservative therapies were ineffective, from January 1, 2023, to October 31, 2023. This trial comprised 106 patients who received modified TFESI alongside PRF. We evaluated clinical efficacy and follow-up at baseline and at 1 week, 1 month, 3 months, 6 months, and 12 months post-surgery primarily using the Visual Analog Scale (VAS), Oswestry disability index (ODI), and modified MacNab score.

**Results:**

Patients who received modified TFESI in conjunction with PRF exhibited substantial enhancements across all three assessment instruments (VAS, ODI, MacNab) when compared to pre-treatment evaluations (*p* < 0.0001). The alleviation of radicular discomfort was notably enduring, meeting the patients’ expectations. At the 12-month follow-up, we noted that patients often achieved substantial pain alleviation within 6 months, and only a minor proportion of patients encountered pain recurrence by the 12th month, with no notable problems detected.

**Conclusions:**

The modified TFESI in conjunction with PRF is a safe, cost-efficient, and successful therapy modality. Our findings indicated that this method can efficiently and swiftly relieve patients’ radicular discomfort and produce enduring therapeutic effects.

## 1 Introduction

Lumbar disc herniation (LDH) is currently one of the most common orthopedic disorders globally. The pathological characteristics include the nucleus pulposus of the lumbar intervertebral disc herniating through the annulus fibrosus, exerting pressure on the nerve roots, or the degeneration of the lumbar intervertebral disc inciting an inflammatory response, resulting in significant clinical manifestations such as radicular pain. LDH impacts 1%–2% of the population, with a global incidence rate of 4.8‰ (1). The annual prevalence of radicular pain due to LDH might attain 2.2% (2). Furthermore, the consequent radiating pain in the lower limbs and prolonged motor dysfunctions considerably diminish patients’ quality of life and may also lead to psychological (3) and social problems (4). Consequently, researchers are increasingly concentrating on mitigating the pain sensations of radiculopathy and enhancing the recovery of lower limb functionality in patients.

A diverse array of therapy modalities exists for radicular discomfort resulting from LDH. This encompasses non-surgical interventions, including physical therapy and medication, in addition to surgical procedures. While non-surgical interventions can effectively mitigate symptoms in most cases (5), a minority of patients continue to suffer from intractable pain or neurological impairments, necessitating additional treatment. Surgical intervention is necessary to alleviate symptoms by removing trauma and local inflammation at the affected spot. Despite the absence of consensus on transforaminal epidural steroid injection (TFESI) in guidelines from various experts due to insufficient class I evidence (6, 7), a substantial multicenter study indicated that TFESI is recommended as the primary treatment for patients with radicular pain resulting from LDH, given the low cost-effectiveness of surgical intervention (8). TFESI administers steroids via injection into the epidural space adjacent to the intervertebral foramina, with the objective of alleviating inflammation and edema surrounding the nerve roots. The analgesic effect of TFESI alone is typically brief and generally necessitates other therapies (9, 10). Pulsed radiofrequency (PRF) is a novel, minimally invasive interventional procedure that is being utilized in the management of patients with radicular pain. It depends on sporadic stimulation using high-frequency electrical currents to induce a neuromodulatory effect, believed to alleviate the sensations of radiating pain in the lower limbs resulting from radicular pain (11). Recent data indicate that TFESI combined with PRF has produced favorable and enduring outcomes in the management of radicular pain resulting from radiculopathy (12). Nonetheless, traditional TFESI and PRF exhibit limitations, including inadequate pain alleviation and ambiguous mid- to long-term effectiveness (13).

Therefore, we advocated the implementation of TFESI in conjunction with PRF, modifying it to improve efficacy. Our team opted to conduct TFESI within the nerve root sheath membrane, utilizing high-voltage and long-duration PRF to treat patients, potentially enhancing therapeutic outcomes such as expedited onset of action, extended pain relief and diminished short- to medium-term recurrence rates. This paper will assess the efficacy of this therapy in addressing lumbar radiculopathy.

## 2 Materials and methods

### 2.1 Study design and inclusion/exclusion criteria

We performed a retrospective observational study to evaluate the effectiveness of modified TFESI in conjunction with PRF for treating lumbar radiculopathy. Patients received physical and neurological assessments. Between January 2023 and October 2023, 106 patients were enrolled based on the established inclusion and exclusion criteria. The criteria for inclusion were as follows:

18 years < Age < 65 years

Clinical presentation:

Pain intensity on VAS ≥ 5
Accompanied with unilateral radiculopathy
Unilateral lower extremity exhibiting radicular pain for more than 3 months.
No prior spinal surgery
All patients received magnetic resonance imaging (MRI) and were diagnosed with mild LDH.
All patients declined open surgery and received ineffective conservative treatment, including physiotherapy and medication (tramadol, NSAIDs, etc.) for three months.

Exclusion criteria were as follows:

Lumbar neoplasms, spinal tuberculosis, lumbar spondylolisthesis, spinal canal stenosis, and lumbar fractures.
Indicators of significant nerve injury encompass motor paralysis, muscle atrophy, and cauda equina syndrome.
Uncontrolled diabetes mellitus, uncontrolled hypertension, cardiovascular disease, malignant neoplasms, and hemorrhagic tendencies.

Multi-segmental LDH or absence of disc herniation.

Infection and coagulopathy.
History of previous epidural injections or medication allergies.
Pregnant individuals and other patients who decline surgical intervention.

### 2.2 Surgical procedure

#### 2.2.1 Modified TFESI combined with PRF

The patient is positioned in a prone stance, with a pillow placed beneath the abdomen to induce slight flexion of the lumbar spine. Utilizing CT guidance, identify the vertebral body level for the block (S1 at the first sacral nerve foramen), and delineate the skin puncture site at the intersection of 4–6 cm lateral to the spinous process and 1.0–1.5 cm medial to the transverse process. Perform routine draping and disinfection, utilizing a 22 G 10 cm or 15 cm puncture needle, targeting the lower third of the transverse process, and under CT guidance, puncture in a forward, upward, and inward direction.

Advance the needle tip 4–6 cm until it contacts the bone, then moderately retract the needle and reorient it to position just beneath the transverse process. Once the bone contact ceases, advance the needle tip until it slightly surpasses the anterior margin of the transverse process. When the needle tip approaches or contacts the nerve root (achieving the target), the patient will experience radiating pain. Inquire whether the symptoms align with the patient's typical manifestations. Upon verification, confirm that the designated location has been attained. Utilize low frequency (2 Hz) and high frequency (50 Hz) sequentially for nerve assessment. The manifestation of muscle twitching and paresthesia in the region of nerve innervation signifies accurate needle placement. Initiate PRF treatment at 80–100 V, 42°C, for a duration of 120–180 s, to be repeated 2–3 times.

**Figure 1:**
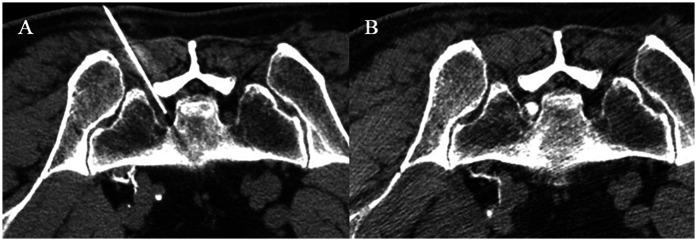
CT-guided modified TFESI combined with PRF. A 58-year-old woman with left S1 nerve root symptoms underwent modified PRF combined with TFESI treatment. **(A)** The puncture needle was inserted through the first sacral foramen to reach the S1 nerve root. After confirming through motor and sensory assessment, PRF treatment was initiated at 80 V and 42°C for a duration of 180 s, repeated three times. **(B)** After the PRF procedure, without withdrawing the puncture needle, slowly advance it into the nerve sheath, inject a small amount of contrast agent, and observe that the contrast agent is confined to the nerve sheath. Without changing the position of the needle tip, inject 1 ml of 1% lidocaine + 1 ml of compound betamethasone.

Upon completion of the PRF procedure, refrain from withdrawing the puncture needle; instead, gently advance the needle into the nerve root sheath and perform a CT scan to verify the needle's placement within the nerve root sheath once more. In the absence of blood or cerebrospinal fluid during withdrawal, a minimal quantity of iohexol may be selectively administered into the target nerve root sheath, ensuring that the diffusion of the contrast agent is confined to the nerve root sheath (Figure 1). Attach the syringe and administer the therapeutic agent (1% lidocaine 1 ml + compound betamethasone 1 ml). Upon completion, withdraw the puncture needle, apply localized pressure to halt bleeding, and cover with a sterile dressing.

### 2.3 Outcome measures

Patient characteristics include baseline information such as sex, age, and symptom duration at the time of admission. Baseline data, comprising the VAS, ODI, and MacNab scores, were gathered before and following the surgical procedure. All evaluations were performed separately by two seasoned physicians.

The VAS is a technique for evaluating the intensity of pain. A 10 cm line serves as an indicator, with one end representing the lack of discomfort and the opposite end denoting the most severe pain. The patient is requested to identify the position on the line that signifies the severity of their pain.

VAS: Scores were recorded before and after surgery. To be defined as effective pain relief, postoperative pain levels must have been reduced by at least 3 points or more (14) from the preoperative period and must have been reduced by 50% compared to the preoperative period (15, 16). Based on the VAS score, our team defined patients with a score of 2 or more points higher than the previous one as having significant pain recurrence as a way of observing pain recurrence during follow-up.

Secondly, the Oswestry Disability Index (ODI) consists of 10 sections, covering pain, individual function, and overall personal function. The lowest score for each item is 0 (good state), and the highest score is 5 (poor state). If all 10 questions are answered, the scoring method is: actual score/50 (maximum possible score) × 100%. If one question is not answered, the scoring method is: actual score/45 (maximum possible score) × 100%. Generally, the higher the score, the more severe the functional impairment is.

The modified MacNab criteria are divided into four grades from excellent to poor. Excellent: Symptoms are completely gone, and the patient has returned to their previous work and lifestyle; Good: There are slight symptoms, with mild limitations in activity that do not affect work or daily life; Fair: Symptoms are reduced, but there are limitations in activity that impact normal work and daily life; Poor: There is no difference before and after treatment, or the condition has even aggravated.

### 2.4 Statistical analysis

Means, standard deviations, medians, quartiles, frequencies, and percentages were used to describe the baseline data. The Mann–Whitney *U* test was employed to analyze the non-normally distributed continuous data before and after surgery. All data were analyzed and graphed using GraphPad Prism 10.2.3 and Windows Office Excel 2019 software.

## 3 Results

### 3.1 Basic characteristics of patients

One hundred six patients received modified TFESI in conjunction with PRF treatment following the ineffectiveness of conservative treatment lasting over three months. Clinical data were gathered pre- and post-procedure, encompassing 48 male patients (45.28%) and 58 female patients (54.72%), aged between 35 and 65 years, with a mean age of 49.92 ± 7.60 years. The general preoperative circumstances encompassed gender, age, duration of preoperative pain, pain location, pain severity, preoperative VAS, and preoperative ODI. All patients participating in the research experienced substantial unilateral radiating leg discomfort (Table 1).

**Table 1:** Basic characteristics and baseline information of patients.

Categorical data	*N* = 106
Age (years)	49.92 ± 7.60
Sex	
Male (%)	48 (45.28%)
Female (%)	58 (54.72%)
Time of radicular pain (months)	10.21 ± 3.45
Herniation segment	
L3-4	14
L4-5	52
L5-S1	40
Symptomatic side	
Left	58
Right	48
Visual analogue scale (VAS)	7.63 ± 0.89
Oswestry disability index (ODI) (%)	67.13 ± 7.80
0%–20%	0 (0%)
21%–40%	0 (0%)
41%–60%	26 (24.53%)
61%–80%	77 (72.64%)
81%–100%	3 (2.83%)

### 3.2 Longitudinal data analysis of the efficacy of modified TFESI combined with PRF

**Figure 2:**
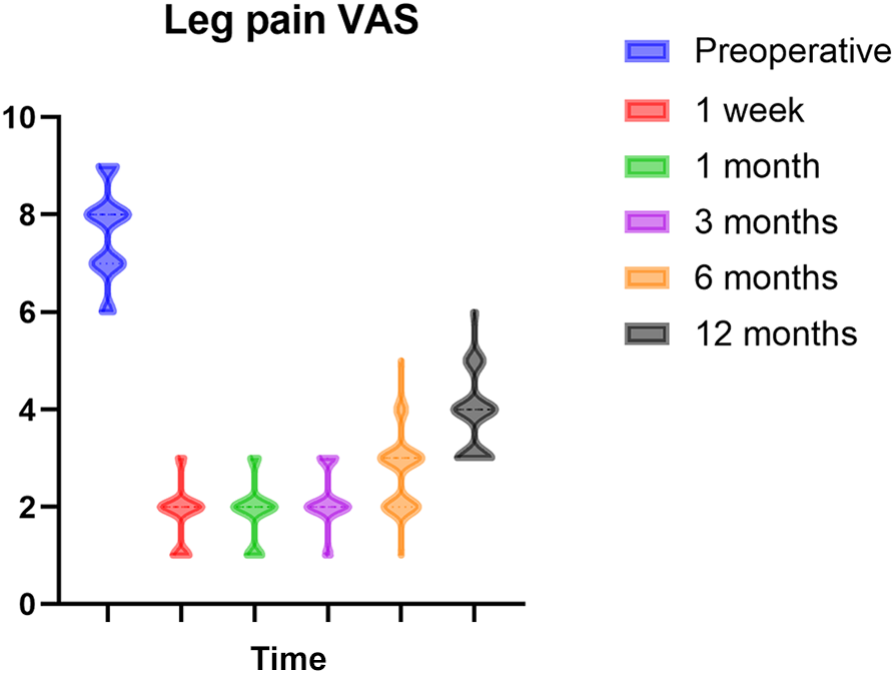
Preoperative and postoperative outcomes measured by leg pain VAS.

The VAS was significantly reduced compared to the baseline VAS at all follow-up assessments (*p* < 0.0001). Patients exhibited notable pain alleviation one week post-surgery. Pain reduction was prompt and effective during the six-month follow-up. In comparison to the VAS results at six months, the scores after 12 months exhibited a modest increase; nonetheless, pain alleviation was adequate relative to preoperative levels. Figure 2 depicts this enhancement. Nonetheless, throughout the follow-up process, some instances of pain recurrence were observed. At six months, five instances of notable pain recurrence were seen, and at twelve months, 27 instances (25.47%) of evident pain recurrence were recorded.

**Figure 3:**
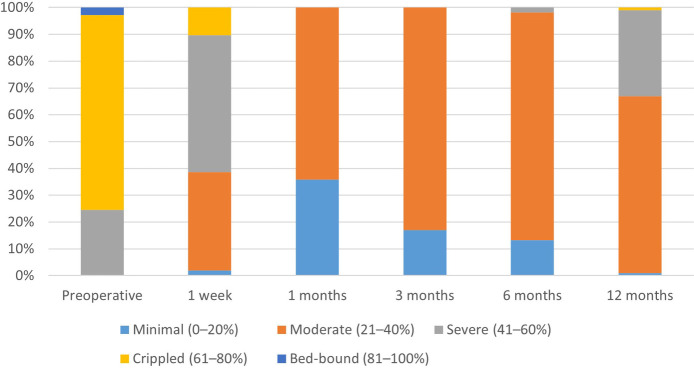
Preoperative and postoperative outcomes measured by ODI.

Table 2 indicates that the postoperative ODI scores were markedly reduced compared to preoperative values (*p* < 0.0001), exhibiting a gradual upward trajectory over time. Figure 3 demonstrates that at six months post-operation, more than 90% of patients attained a functional level that minimally affected their daily lives. At 12 months post-operation, almost 60% of patients continued to experience minimal effects on their everyday activities. This suggests that the integrated treatment can enhance both the quality of life and the restoration of physical functions.

**Table 2:** Preoperative and postoperative outcomes measured by ODI.

Level of disability/time	Preoperative	1 week	1 month	3 months	6 months	12 months
Minimal (0%–20%)	0 (0%)	2 (1.89%)	38 (35.85%)	18 (16.98%)	14 (13.21%)	1 (0.94%)
Moderate (21%–40%)	0 (0%)	39 (36.79%)	68 (64.15%)	88 (83.02%)	90 (84.90%)	70 (66.04%)
Severe (41%–60%)	26 (24.53%)	54 (50.94%)	0 (0%)	0 (0%)	2 (1.89%)	34 (32.08%)
Crippled (61%–80%)	77 (72.64%)	11 (10.38%)	0 (0%)	0 (0%)	0 (0%)	1 (0.94%)
Bed-bound (81%–100%)	3 (2.83%)	0 (0%)	0 (0%)	0 (0%)	0 (0%)	0 (0%)
*p* value		<0.0001	<0.0001	<0.0001	<0.0001	<0.0001

The assessment outcomes based on the modified MacNab criteria (Table 3) indicate that the rates of excellent and good results in the initial six months are above 90%, with the excellent rate exhibiting a gradual rising trajectory. By the twelfth month, the excellent and good rate decreased to 72.64%. The symptoms of certain patients evidently recurred, adversely impacting their normal life capacities to some degree.

**Table 3:** Postoperative outcomes measured by modified MacNab criteria.

Time	Excellent	Good	Fair	Poor	Excellent rate	Good and excellent rate
1 week	17	89	0	0	16.04%	100%
1 month	30	76	0	0	28.30%	100%
3 months	33	73	0	0	31.13%	100%
6 months	30	69	7	0	28.30%	93.40%
12 months	10	67	29	0	9.43%	72.64%

## 4 Discussion

LDH is a prevalent spinal condition characterized by the protrusion of the nucleus pulposus, which frequently results in intense radicular pain due to mechanical compression, neuroinflammatory responses, or localized ischemia. This disorder adversely impacts the patient's quality of life and may result in enduring misery and potential impairment. The management of radicular pain includes both conservative and surgical interventions. Evidence indicates that early minimally invasive interventional treatments may outperform long-term conservative therapy regarding pain alleviation and functional recovery (17). Minimally invasive interventional therapies, characterized by reduced trauma and expedited recovery, are increasingly recognized as a significant option for addressing lumbar radiculopathy resulting from LDH (18). Nonetheless, each minimally invasive interventional treatment possesses inherent limitations, and the duration of therapeutic benefits exhibits considerable variability. Monotherapy may be less efficacious; hence, combination therapy frequently fulfills the objective of prolonging the therapeutic impact.

Epidural steroid injection (ESI) is often performed using three techniques: interlaminar epidural steroid injection (ILESI), TFESI, and caudal epidural steroid injection (CESI). TFESI has emerged as a prevalent minimally invasive interventional treatment for addressing radicular pain as a result of lumbar radiculopathy (6). Gharu et al. (19) demonstrated that there was no significant disparity in therapeutic outcomes between ILESI and TFESI within six months following treatment. Conversely, Lee et al. (20) contended that TFESI surpasses ILESI for both efficacy and safety, aligning closely with the experimental findings of Haring et al. (21). These investigations revealed the superiority of TFESI in target specificity, particularly indicating enhanced clinical efficacy compared to ILESI in the treatment of radicular pain. A prospective research showed that both CESI and TFESI effectively reduce pain and impairment in patients with unilateral S1 radiculopathy (22). Given that our study concentrates on lumbar radiculopathy, we have judiciously chosen TFESI as one of the therapeutic approaches. Wilby et al. (8) demonstrated that, following 12 months post-operation, TFESI and lumbar microdiscectomy had comparable efficacy in alleviating radicular pain caused by lumbar radiculopathy in their study. Concurrently, they endorsed TFESI for the management of radicular pain. TFESI entails the insertion of a needle into the intervertebral foramen at the spinal level to accurately administer drugs, including steroids and local anesthetics, to the vicinity of a particular nerve root. This strategy primarily provides pain relief by allowing drugs to exert anti-inflammatory and analgesic effects, modify or disrupt nociceptive input, and influence the reflex processes of afferent fibers (23). A comprehensive meta-analysis indicated that TFESI effectively alleviated radicular pain attributed to lumbar radiculopathy during a six-month period; however, its long-term analgesic efficacy was constrained (10). TFESI serves as an interventional treatment that can successfully alleviate pain and restore physiological functioning in the medium to short term; however, it does not seem to serve as a long-term alternative to surgical intervention.

PRF is utilized in the management of many forms of neuropathic pain, including radicular discomfort resulting from lumbar radiculopathy. The objective of PRF therapy is to attain sustained analgesia and safeguard the nerves from injury. It generates pulsed stimuli that regulate nerve activity and can alleviate pain resulting from nerve injury and mechanical compression (24). Recent studies indicate that PRF increases the production of anti-inflammatory factors, including Na/K-ATPase and 5-HT3r, while simultaneously suppressing pro-inflammatory factors such as TNF-α and IL-6, hence diminishing the neurogenic inflammatory response (25). It also suppresses the activation of the MAPK pathway in the spinal cord, diminishes the release of cytokines and excitatory amino acids (26), inhibits the activation of the JNK pathway in the dorsal horn of the spinal cord (27), and prevents spinal cord sensitization, leading to a reduction in acute pain and an enhancement of neuropathic pain (28). A research involving 149 patients with trigeminal neuralgia who had PRF treatment demonstrated a median recurrence-free interval of up to 118 months (29). The findings demonstrate that PRF treatment can provide effective pain alleviation for an extended duration. However, as time goes, the recurrence rate will continue gradually climb. PRF treatment may require integration with additional therapeutic modalities to attain sustained pain management. This treatment method primarily depends on the physical electrical stimulation produced by PRF, which influences neural pathways and modulates cytokines to achieve a therapeutic effect. In recent years, TFESI in conjunction with PRF has been extensively utilized for radicular pain in clinical settings. While certain studies (30) indicate no significant difference in the reduction rate of radicular pain between combined treatment and PRF alone, the majority of studies assert that the combined therapy offers advantages over monotherapy, particularly in achieving higher levels of pain relief and a relatively prolonged duration (12, 13, 31). In a randomized controlled study, the results showed that compared with the use of TFESI alone, the combination of PRF and TFESI provided better pain relief within 3 months (32). This indicates that the treatment with the combination of PRF and TFESI can enhance the efficacy and response rate of steroid injection. Compared to the patients who received PRF treatment alone, the patients in the TFESI combined with PRF group had the lowest level of pain and the best improvement in the ODI one month after the surgery. In addition, a retrospective study by Ding et al. (24) involving 135 patients with lumbar radiculopathy demonstrated that the combination of PRF and TFESI for the treatment of lumbar radiculopathy could significantly reduce the VAS score and the ODI in the early postoperative period. Moreover, this effect remains superior to that of patients who received PRF or TFESI alone even six months after the surgery. The satisfaction level of patients in the PRF combined with TFESI group is also dramatically higher than that of patients who received PRF or TFESI alone. This indicates that the combined treatment can indeed more effectively improve the symptoms and quality of life of patients. The combined treatment, utilizing the simultaneous action of pharmaceuticals and PRF, may yield effective short-term analgesia. Patients who received the combined treatment rarely experienced severe adverse events and had a reduced use of painkillers. In addition, this surgery has less trauma compared to traditional surgeries, with minimal damage to muscles and nerves. As a result, it reduces the incidence of complications and cuts down on costs (31). Consequently, our research team is dedicated to enhancing this combined therapy for lumbar radiculopathy.

The majority of published research on TFESI and radicular pain has chosen to administer medicines around the nerve root. Our team has altered this method by opting to conduct TFESI within the nerve root sheath. This technique can precisely target the nerve root affected by compression while preserving adjacent healthy nerve tissues (33). Conversely, administering TFESI near the nerve root may adversely affect adjacent healthy tissues. Secondly, the direct injection of the drug into the nerve root sheath addresses the limitation of typical TFESI, which fails to provide an adequate concentration of corticosteroids to the location of nerve root irritation (34), hence facilitating more effective alleviation of radicular pain. Roberts et al. (35) demonstrated in their review that intraneural steroid injection is more effective than perineural root block for alleviating radicular pain resulting from lumbar radiculopathy. Viswanathan et al. (36) reviewed that intraneural steroid injection can offer patients both short- and long-term pain alleviation. A study on the safety of intraneural injections indicated that low-volume intraneural injections did not result in substantial harm (37). Recent research indicates that the optimal placement of the needle tip for nerve block is situated between the innermost layer of the epineurium and the epineurium itself, facilitating effective outcomes without causing nerve injury (38). The studies aforementioned indicate that intraneural sheath TFESI is an efficacious approach for alleviating radicular pain, providing substantial symptom reduction in the medium to short term, accompanied by a low short-term recurrence rate, favorable tolerability, and safety for radicular pain management. Consequently, our revised TFESI demonstrates notable gains regarding precision, efficacy, safety, and the minimization of surgical intervention over time.

Secondly, there is no universally ideal parameter for PRF therapy. A study report indicates that a temperature of 42°C is believed to preserve motor nerve function while offering substantial efficacy for neuralgia in comparison to conventional continuous radiofrequency (CRF) treatment (39). The standard PRF parameters include an electrode tip temperature of 42°C, an output voltage of 45 V, and a treatment duration of 120 s (40). Tanaka et al. (41) discovered in an animal model study that extending the PRF exposure duration from 2 min to 6 min markedly improves the efficacy of PRF in alleviating neuropathic pain. In recent years, a high-voltage and prolonged-duration PRF mode has been developed to enhance therapy efficacy. The treatment parameters are established at a temperature of 42°C at the electrode tip, an output voltage ranging from 50 to 90 V, and a duration of 900 s, which can yield good analgesic results without significant problems (42). Wang et al. (43) also posited that an extension of treatment duration correlates with enhanced pain relief. Our team has opted to adjust the conventional PRF treatment parameters based on prior professional research. We administered a high-voltage, prolonged PRF treatment to patients, establishing the parameters at 42°C, a voltage range of 80–100 V, and a duration between 240 s and 900 s, contingent upon the desired effect as reported by the patients post-procedure. During the 12-month follow-up after our modified combined treatment, over 90% of patients experienced good pain relief within 6 months, and less than 5% of the cases had obvious pain recurrence. More than 60% of patients maintained good pain relief within 12 months, while the recurrence rate reached 25%. Compared to previous literature, the study by Karakose et al. (44) indicates that among 67 patients who underwent PRF combined with TFESI treatment, the recurrence rate requiring subsequent surgery was as high as 25.8% just three months post-operation, whereas the recurrence rate of our modified combined treatment is significantly lower than this result. Despite several patients exhibiting significant pain recurrence 12 months post-surgery, their functional level, as indicated by the ODI score, surpassed that of patients undergoing standard combination treatment (31). This suggests that the altered combined treatment provides effective pain relief and a low recurrence rate in the short to medium term; however, there remains a risk of recurrence in the long term.

In conclusion, our research chooses to manage patients with lumbar radiculopathy with a modified approach combining TFESI and PRF. This treatment can effectively and sustainably relieve severe radicular pain in the lower limbs of patients, enabling a swift return to normalcy. Our research findings further validated that the modified TFESI, combined with PRF, had a rapid onset, prolonged effects, and a minimal recurrence rate in both the medium and short term. It also significantly enhances patients’ quality of life and increases their satisfaction.

## 5 Limitations

The limitation of our study is that it was a single-center retrospective study and lacked a control group. This study aimed to investigate the effectiveness of modified TFESI combined with PRF in the treatment of radicular pain based on clinical data. The long-term effects of this therapy as well as the rate of distant recurrence are issues that require further research in the future.

## 6 Conclusion

The modified TFESI combined with PRF is a minimally invasive interventional technique. We discovered that it could yield lasting and substantial enhancement in the management of radicular pain throughout an average follow-up duration of 12 months. In conclusion, this suggests that this minimally invasive surgery is a viable method and may yield superior outcomes in achieving medium- or long-term pain management. Additional rigorously controlled, prospective studies with adequate sample numbers are required to validate these results.

## Data Availability

The raw data supporting the conclusions of this article will be made available by the authors, without undue reservation.
